# Microbial enzyme-driven carbon-nitrogen cycling processes of greenhouse gases in sandy sediments of river-oasis systems in arid regions

**DOI:** 10.3389/fmicb.2026.1912116

**Published:** 2026-09-03

**Authors:** Guoqing Lv, Junjian Wang, Lijun Hou, Xigang Liu, Mao Ye, Xiaoshuang Zhao, Yaqi Song, Zongxiao Zhang, Yonghui Wang

**Affiliations:** 1 College of Geography Science and Tourism, Xinjiang Normal University, Urumqi, China; 2 State Key Laboratory of Soil Pollution Control and Safety, MEE Environmental Protection Key Laboratory of Integrated Surface Water-Groundwater Pollution Control, Guangdong Provincial Key Laboratory of Soil and Groundwater Pollution Control, School of Environmental Science and Engineering, Southern University of Science and Technology, Shenzhen, China; 3 State Key Laboratory of Estuarine and Coastal Research, East China Normal University, Shanghai, China; 4 Xinjiang Laboratory of Lake Environment and Resources in Arid Zone, Xinjiang Normal University, Urumqi, China; 5 Technical Research Center for Environmental Geotechnical Engineering Restoration and Resource Utilization, Xinjiang Normal University, Urumqi, China

**Keywords:** biogeochemical hotspots, carbon–nitrogen transformation, greenhouse gas production, microbial extracellular enzymes, oasis agricultural area, sediment texture heterogeneity

## Abstract

Arid inland rivers are important components of the land–water–ocean continuum, yet the linkages among sediment texture, microbial-mediated processes, and greenhouse gas (GHG) production remain poorly understood in sandy systems. Here, we investigated surface (0–5 cm) and subsurface (5–10 cm) sediments from the Niya River Basin, a desert–oasis agricultural river in northwestern China, by integrating sediment particle-size characterization, physicochemical measurements, extracellular enzyme assays, GHG production experiments, and multivariate analyses. We examined how sediment texture and carbon–nitrogen substrate availability regulate enzyme activities and CO₂, CH₄, and N₂O production potentials. Sediments were dominated by sand (>78%) but exhibited pronounced spatial heterogeneity in enzyme activities and GHG production. Localized hotspots of enzyme activities and CH₄/N₂O production were associated with enriched TOC, TN, and NH₄^+^. Multivariate analyses revealed depth-dependent environmental associations: surface processes were primarily linked to TOC and pH, whereas very coarse sand showed increased relative importance in subsurface sediments, particularly for GHG production. These findings demonstrate that sandy sediments in arid inland rivers are not inert environments; rather, carbon–nitrogen substrate availability and particle-size heterogeneity jointly shape microbial enzyme-mediated transformation processes and spatially variable GHG production potentials.

## 1 Introduction

Arid inland rivers, which provide essential water resources for oasis agriculture and sustain the ecological stability of desert–oasis systems, have become increasingly important components of the land–water interface carbon and nitrogen cycles. But current research lacks a systematic understanding of this phenomenon (Ramond et al., 2022). Under ongoing climate change, these systems are increasingly recognized not as passive transport corridors, but as active biogeochemical reactors that regulate the transformation, retention, and export of carbon and nitrogen from inland drylands to downstream aquatic environments (Chanu et al., 2022). Inland waters simultaneously function as both sedimentary carbon reservoirs and episodic sources of GHGs (CO₂, CH₄, and N₂O), thereby exerting significant control on regional and global carbon budgets. Moreover, intermittent riverine export of dissolved and particulate organic matter provides an important but often underappreciated linkage between arid inland catchments and downstream aquatic–coastal systems, thereby coupling terrestrial dryland processes with broader hydrospheric carbon dynamics (Dong et al., 2023; Qin et al., 2025). Despite this recognition, the internal processes controlling carbon and nitrogen transformation in river sediments remain poorly elucidated, particularly in arid river systems where hydrological intermittency, intense evaporation, and spatially heterogeneous sediment environments can lead to highly variable biogeochemical conditions (Geng et al., 2025). Compared with perennial rivers in humid regions, arid inland rivers are characterized by limited precipitation, intense evaporation, seasonal runoff variability, weak hydrological connectivity, and frequent wet–dry transitions (Datry et al., 2023). These conditions can strongly alter sediment transport, salinity accumulation, particle-size sorting, organic matter retention, and microbial habitat structure. And the intermittent flow and episodic rewetting may further promote pulsed biogeochemical activity, allowing dry or seasonally exposed sediments to act as temporary but active zones of carbon and nitrogen transformation (Cui et al., 2024). In such systems, localized carbon and nitrogen enrichment, fine-particle-size retention, and heterogeneous moisture conditions may provide favorable microsites for organic matter decomposition and nutrient transformation (Cates et al., 2022). The interaction between substrate availability and sediment particle-size can further regulate particle-size connectivity, water retention, oxygen diffusion, and microscale redox gradients (Chao et al., 2026). Therefore, carbon–nitrogen substrates and sediment texture may jointly shape the microsites where microbial functional processes, extracellular enzyme activity, and greenhouse-gas production rates.

River sediments are among the most active interfaces for carbon-nitrogen cycling (Chen et al., 2024). Within these sedimentary systems, microbial processes constitute the fundamental engine driving carbon-nitrogen cycling. Extracellular enzymes, as key functional products of microbial communities, mediate the breakdown of complex organic matter and regulate the conversion of particulate and dissolved organic carbon and nitrogen into bioavailable substrates. Accordingly, extracellular enzyme activity should be interpreted as a direct functional expression of microbial metabolic potential, rather than a simple environmental indicator. It integrates microbial community function with sediment biogeochemical transformation processes, thereby providing a mechanistic link between substrate availability and ecosystem metabolism. They serve not only as temporary reservoirs of particulate organic matter, nitrogen, and microbial communities, but also as hotspots of organic carbon mineralization, nitrogen transformation, methane production, and nitrous oxide generation (Cabugao et al., 2022). The production pathways of different GHGs are gas-specific. CO₂ production is generally associated with widespread organic carbon mineralization and can occur across a broad range of redox conditions (Kim et al., 2023). CH₄ production is more dependent on localized anaerobic or reducing microenvironments and sufficient bioavailable organic substrates (Wu et al., 2021), whereas N₂O production is closely linked to nitrification, denitrification, and redox transitions at oxic–anoxic interfaces (Han et al., 2023). Sediment extracellular enzymes provide a functional bridge between substrate supply and microbial metabolism by catalyzing the degradation of organic matter and nutrient release. Enzyme activities are affected by organic matter availability, nutrient status, pH, moisture conditions, mineral substrates, and sediment microenvironmental heterogeneity (Batista et al., 2022). Thus, sediment enzyme activity was understood as an integrated functional response rather than as the direct outcome of a single nutrient factor. Currently, research on sediment enzymatic activity and GHG production primarily focuses on lakes, wetlands, estuaries, agricultural sediments, and river sediments in humid regions (Ren et al., 2026; Zhu et al., 2025). These environments commonly have higher moisture availability, greater fine-particle-size accumulation, more continuous organic matter deposition, and relatively stable redox gradients, which favor organic matter preservation and anaerobic metabolism. By contrast, sediments in arid seasonal inland rivers are often sandy, alkaline, saline, and weak in organic matter retention, but may still contain localized fine-particle-size and substrate-enriched patches (Humphries and McCarthy, 2022). Importantly, sediment particle-size composition showed strong associations with microbial-mediated processes, suggesting that sediment physical structure may potentially influence microscale conditions, including oxygen diffusion, water retention, and redox heterogeneity. Therefore, sediment biogeochemical functioning may be conceptualized as an emergent property arising from the interactions among microbial enzymatic activity, substrate availability, and physical habitat structure.

The Niya River Basin, located on the southern margin of the Tarim Basin in northwestern China, represents a typical desert–oasis agricultural river system and provides an ideal natural laboratory for investigating sediment biogeochemical processes under extreme arid conditions (Yang et al., 2006). The basin is sustained by snow and glacier meltwater from the Kunlun Mountains and supports limited but highly concentrated oasis agriculture within an otherwise hyper-arid desert matrix. It therefore constitutes a typical mountain–oasis–desert continuum, where agricultural oases act as key zones of intensified land–water interaction, vegetation concentration, and anthropogenic nutrient input. In this system, hydrological variability and long-term climatic fluctuations may influence sediment transport processes and contribute to variations in ecosystem stability (Shi et al., 2026). Importantly, agricultural activity within oasis zones may further modify sediment carbon and nitrogen inputs, thereby enhancing microbial activity and altering enzyme-mediated biogeochemical processes. However, whether arid sandy river sediments, often considered to have limited biogeochemical reactivity, can sustain localized zones of enhanced microbial activity remains poorly understood. In this study, we investigated whether spatial heterogeneity in sediment texture and carbon–nitrogen availability was associated with the distribution of biogeochemical hotspots and variations in greenhouse gas production through potential links with microbial enzyme activities. We hypothesized that, despite overall nutrient limitation in sandy sediments, localized differences in sediment properties and substrate availability may generate heterogeneous microscale conditions favorable for microbial activity. Furthermore, we aimed to characterize hotspot distribution and examine potential pathways connecting sediment physical properties, substrate availability, enzyme activities, and CO₂, CH₄, and N₂O production.

## 2 Materials and methods

### 2.1 Study area overview

**Figure 1:**
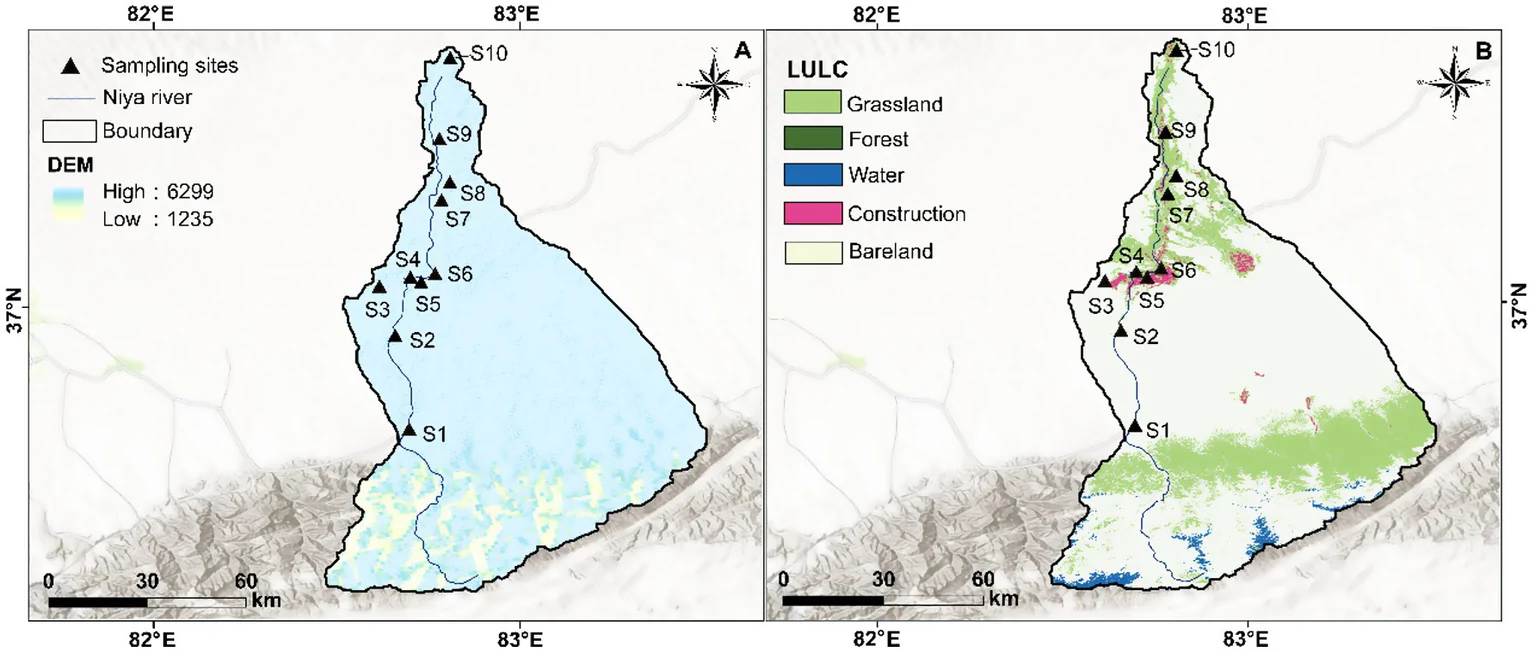
Geographic location and environmental background of the Niya River Basin. **(A)** Digital elevation model (DEM) showing the topographic setting of the study area and the distribution of sampling sites along the Niya River. The black triangles represent sampling sites, the blue line indicates the Niya River, and the black boundary outlines the study area. **(B)** Spatial distribution of land-use/land-cover (LULC) types within the study area, classified as grassland, forest, water, built-up land, and barren land.

The Niya River Basin is located on the northern flank of the Kunlun Mountains and along the southern margin of the Tarim Basin in southern Xinjiang, China (Zhong et al., 2007), representing a typical seasonal inland river system under an arid to hyper-arid climate (Figure 1). Importantly, it contains desert–oasis agricultural areas that are key production zones in southern Xinjiang, relying on meltwater for irrigation and forming a strong mountain–oasis–desert gradient that controls hydrological connectivity, sediment transport, and organic matter inputs (Peng et al., 2023). Under conditions of low precipitation, high evaporation, and frequent wet–dry alternation (Yang et al., 2004), this system develops highly heterogeneous sediments with localized carbon and nitrogen enrichment driven by both natural processes and agricultural activity. In this context, extracellular enzymes represent the fundamental biochemical basis of microbial carbon-nitrogen cycling, and their activity reflects the functional expression of sediment microbial communities, linking substrate availability with biogeochemical transformation processes in arid river sediments.

### 2.2 Sediment sampling and sample preparation

Sampling was conducted in the Niya River Basin, a typical arid inland river system in southern Xinjiang, China. Surface (0–5 cm) and subsurface (5–10 cm) sediments were collected at 10 sampling sites along the main river channel during the dry season. At site S3, the subsurface sediment (5–10 cm) could not be collected due to the extremely coarse, poorly consolidated sandy substrate, which caused borehole collapse and groundwater seepage during excavation, preventing intact core recovery and thus excluding this site from depth comparisons. Each sampling site was geo-referenced using a handheld GPS (±3 m accuracy). At each site, overlying plant residues and debris were carefully removed before sampling to minimize external contamination. Sediments were collected using a stainless-steel auger and transferred into pre-cleaned polyethylene bags. After collection, sediment samples were stored under low-temperature and dark conditions and transported to the laboratory as soon as possible. To ensure representativeness, three subsamples were collected within a 5 m radius and homogenized into one composite sample per depth. Samples were immediately stored in insulated boxes with ice packs (≈4 °C) and transported to the laboratory within 24 h. In the laboratory, each sample was thoroughly homogenized and divided into subsamples for the determination of physicochemical properties and particle-size composition, enzyme activity assays, and GHG production-rate measurements (Attademo et al., 2021; Bastida et al., 2021). All samples were pre-processed following a standardized protocol before analysis to ensure comparability across measured variables.

### 2.3 Measurement of sediment physicochemical properties and particle-size distribution composition

Sediment physicochemical characterization included measurements of pH, electrical conductivity (EC), moisture content (MC), salinity, temperature (T), total carbon (TC), total organic carbon (TOC), total nitrogen (TN), and ammonium nitrogen (NH₄^+^). pH and EC were used to represent sediment acid–base status and soluble salt background, respectively. TC, TOC, TN, and NH₄^+^ were used as indicators of sediment carbon and nitrogen substrate availability, while MC and salinity represented sediment moisture status and saline conditions. All physicochemical parameters were measured using standard sediment analytical protocols, and sampling sites were treated as the basic statistical units in subsequent analyses (Pandion et al., 2022). Sediment particle-size composition was determined to characterize spatial differences in sediment particle-size structure among sampling sites. The particle-size fractions were grouped into three major classes—clay, silt, and sand—and further divided into very fine clay, fine clay, medium clay, coarse clay, very fine silt, fine silt, medium silt, coarse silt, very fine sand, fine sand, medium sand, coarse sand, and very coarse sand. The total proportions of clay, silt, and sand were calculated for each site based on the percentage contribution of individual fractions. Representative particle-size metrics, including D50, mean particle-size, and very coarse sand content, were further extracted for Mantel tests, NMDS analysis and random forest-based importance analysis.

### 2.4 Determination of microbial enzyme activities and GHG production rates

To characterize sediment biochemical processes associated with organic matter decomposition, nitrogen transformation, and redox regulation, we measured four enzyme activities: urease, catalase, polyphenol oxidase, and acid protease. Urease and acid protease were used as indicators of organic nitrogen hydrolysis and nitrogen transformation potential (Abdo et al., 2022), catalase reflected the oxidative regulation capacity of sediment organic matter (Attademo et al., 2021), and polyphenol oxidase was associated with the oxidative degradation of aromatic and structurally complex organic compounds (Sinsabaugh, 2010). Field sampling, laboratory measurements, and statistical preprocessing were integrated to quantify microbial enzyme activities and GHG production rates in surface and subsurface sediments. Enzyme activity data were compiled at the sampling-site level, and independent enzyme activity matrices were constructed for the two sediment layers. Before multivariate analyses, all enzyme activity variables were standardized to eliminate the effects of differences in measurement units and numerical scales.

Enzyme activities were determined using substrate-specific colorimetric assays. Sediment samples were incubated with specific substrates, and enzyme activities were quantified based on the absorbance changes of reaction products. Urease activity was measured using 0.10 g sediment incubated with urea solution and citrate buffer at 37 °C for 24 h, followed by colorimetric determination of released ammonium at 578 nm. Catalase activity was determined by incubating 0.05 g sediment with hydrogen peroxide solution for 20 min and measuring residual hydrogen peroxide at 240 nm. Polyphenol oxidase activity was assessed after incubation of 0.05 g sediment with pyrogallol solution at 30 °C for 2 h, with absorbance measured at 430 nm. Acid protease activity was determined using casein as the substrate after incubation at 40 °C for 30 min, followed by Folin–Ciocalteu color development at 680 nm. Substrate-free controls and reagent blanks were included to correct for background signals. Absorbance measurements were performed using a full-wavelength microplate reader (MD190). Enzyme activities were expressed as μg g^−1^ h^−1^ for urease and acid protease, μmol g^−1^ d^−1^ for catalase, and mg g^−1^ h^−1^ for polyphenol oxidase.

Sediment GHG production rates were determined for CO₂, CH₄, and N₂O using a closed-incubation approach modified (González-Piana et al., 2025). 10 g of fresh surface and subsurface sediment samples were placed separately into gas-tight incubation bottles and incubated in the dark for 24 h without external substrate addition. Three parallel incubation bottles and sediment-free blank controls were prepared for each sample. After incubation, headspace gases were collected with gas-tight syringes, and CO₂, CH₄, and N₂O concentrations were quantified by gas chromatography after calibration with standard gas mixtures. The three gases were measured using thermal conductivity, flame ionization, and electron capture detectors, respectively. Production rates were calculated from changes in headspace gas concentrations during incubation, with corrections for blank accumulation, headspace volume, and sediment dry mass. The production rates of CO₂, CH₄, and N₂O were expressed as μg kg^−1^ d^−1^ dry sediment.

### 2.5 Statistical analysis

**Figure 2:**
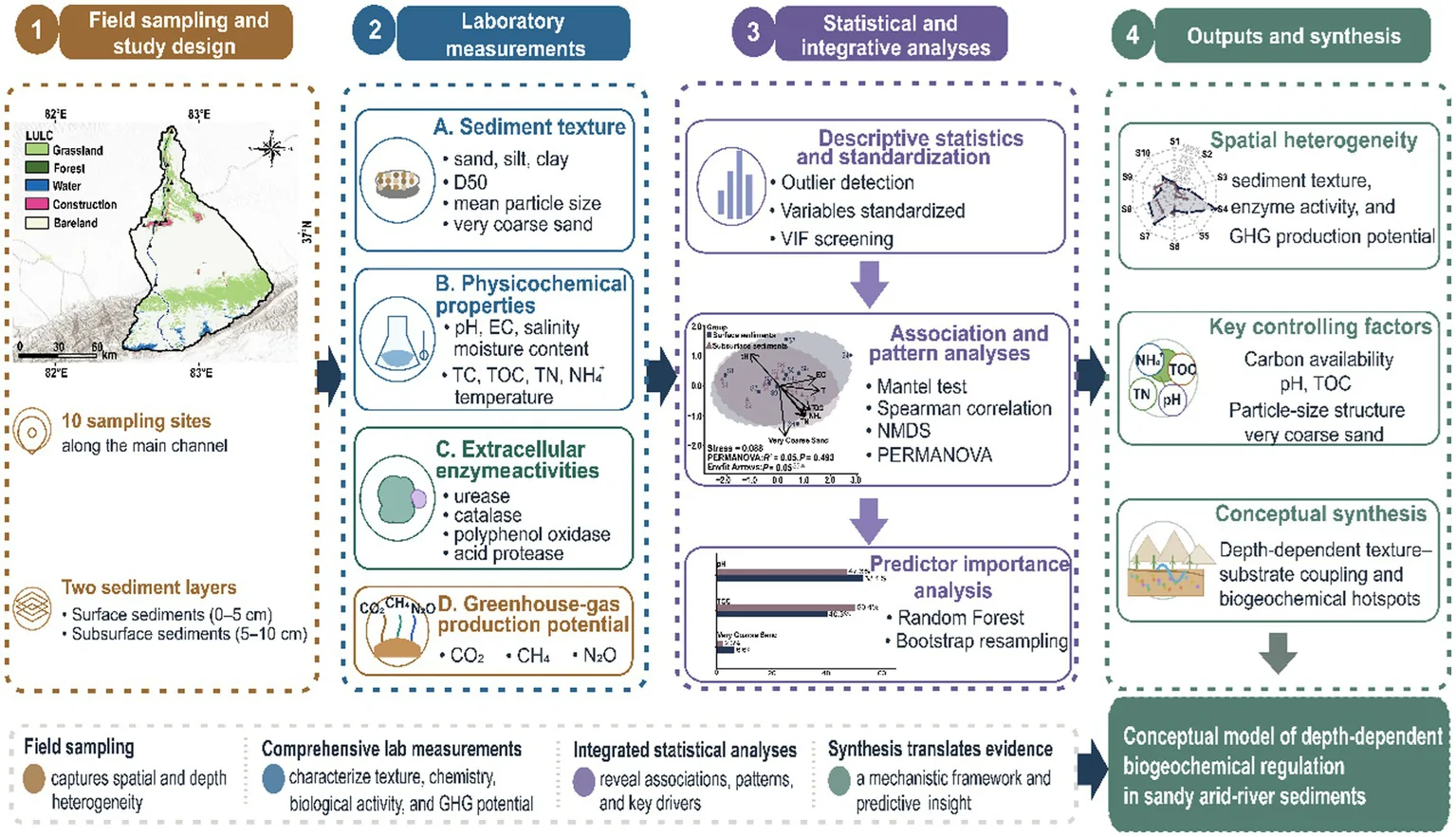
Conceptual framework illustrating potential texture–substrate interactions associated with biogeochemical hotspots in sandy sediments of an arid inland river. EC, electrical conductivity; TC, total carbon; TOC, total organic carbon; TN, total nitrogen; NH_4_^+^, ammonium nitrogen; GHG, greenhouse gases.

Missing values and outliers were removed before statistical analysis. Because 5–10 cm sediment samples were unavailable at site S3, this site was excluded from paired surface–subsurface comparisons. Descriptive statistics were used to summarize spatial variations in sediment particle-size composition, physicochemical properties, enzyme activities, and GHG production rates. All variables used in multivariate analyses were standardized prior to analysis (Ge et al., 2021). Mantel tests were conducted to assess matrix-level relationships between enzyme activities, GHG production rates, and environmental variables. Non-metric multidimensional scaling (NMDS) combined with PERMANOVA was used to visualize differences among samples and evaluate sediment layer effects. Spearman correlation analysis was performed to examine pairwise relationships among individual variables. Variance inflation factor (VIF) analysis was applied to identify and remove highly collinear predictors before random forest analysis. Random forest models were subsequently used as an exploratory approach to rank the relative importance of environmental predictors associated with enzyme activities and GHG production rates. To evaluate the stability of variable importance estimates under the limited sample size, each random forest model was repeated using bootstrap resampling, and the mean importance and standard deviation were calculated. All statistical analyses were performed in R, with statistical significance defined as *p* < 0.05 (Figure 2).

## 3 Results

### 3.1 Spatial patterns of sediment particle-size composition, physicochemical characteristics, enzyme activities, and GHG production rates

**Figure 3:**
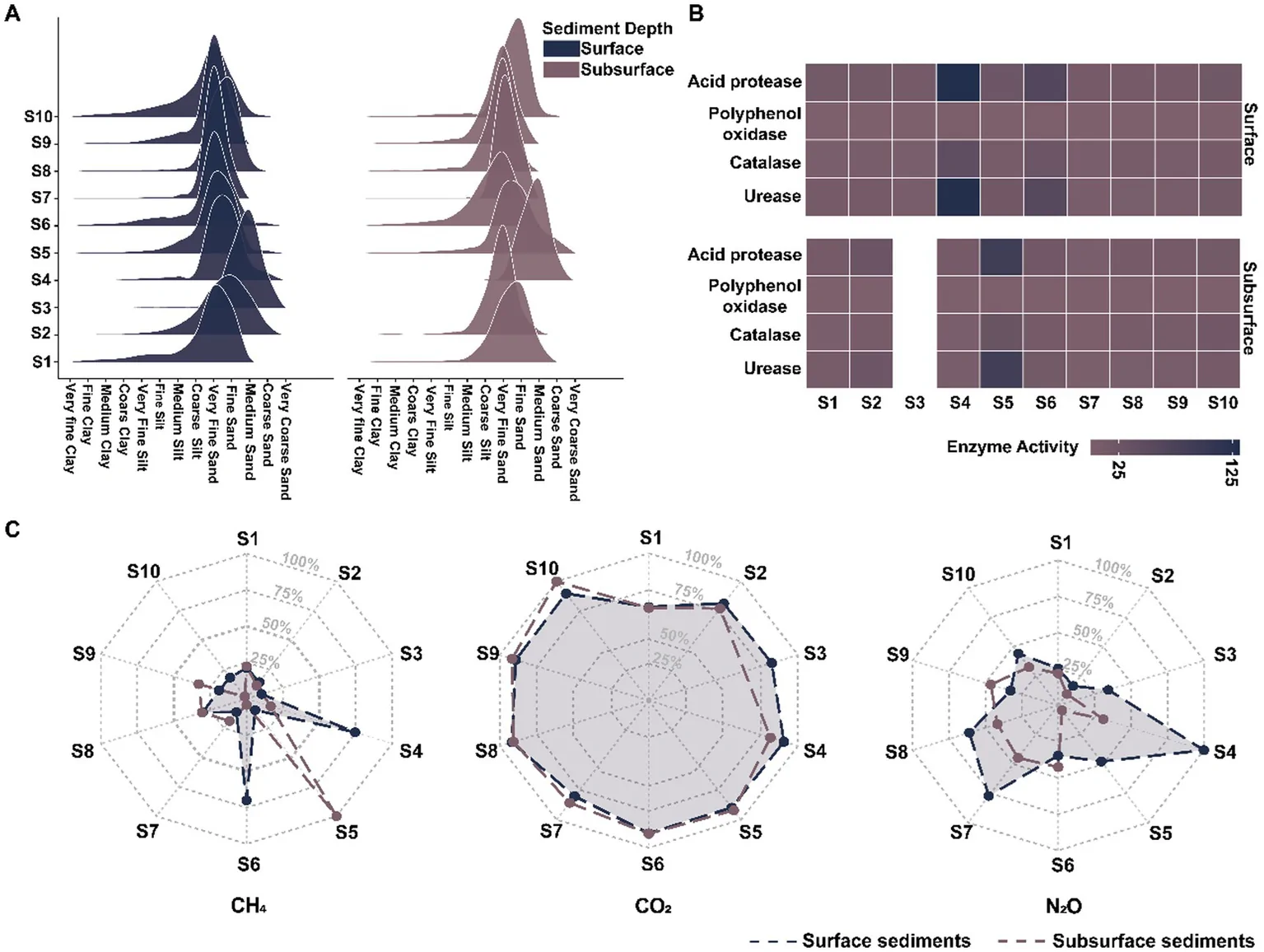
Spatial heterogeneity of grain-size composition, enzyme activity, and GHG production rates in surface and subsurface sediments of the Niya River Basin. **(A)** Displays the peak distribution of major sediment grain-size fractions across different sampling sites and depths. **(B)** Presents heatmaps illustrating the activities of four extracellular enzymes (including acid protease, catalase, polyphenol oxidase, and urease). **(C)** Displays radar charts showing the normalized production rates of CH₄, CO₂, and N₂O, respectively. Blue and purple colors represent surface and subsurface sediments, respectively. Due to field constraints on subsurface sediments collection at some sites, the 5–10 cm sediment sample was not available at site S3. Such data were excluded from depth-paired comparisons.

Collectively, sediment particle-size composition, physicochemical properties, enzyme activities, and GHG production rates revealed a spatial pattern of sediment processes characterized by a dominant sandy background but pronounced local heterogeneity in the Niya River Basin. Both surface sediments (0–5 cm) and subsurface sediments (5–10 cm) were dominated by sand fractions, with sand contents ranging from 60.50 to 97.95% and 52.99 to 98.01%, and mean values of 79.41 and 78.22%, respectively. Although silt and clay accounted for relatively small proportions, their contents varied substantially among sampling sites, indicating that the basin sediments were broadly governed by coarse-grained deposition but locally modified by fine-particle-size accumulation. In surface sediments, S3 showed the highest sand content and the most pronounced coarse-grained signature, whereas S10 exhibited relatively elevated silt and clay contents. In subsurface sediments, the highest sand content occurred at S4, while S6 was characterized by marked silt enrichment, suggesting clear among-site differences in hydrodynamic sorting and depositional settings (Figure 3A). Physicochemically, sediments from both layers were predominantly alkaline (Supplementary Tables S1, S2), with pH ranging from 8.11 to 9.65 in the surface layer and from 8.44 to 9.73 in the subsurface layer. EC, salinity, and carbon–nitrogen nutrient indicators also varied substantially among sites. High TOC, TN, and NH₄^+^ values in surface sediments were mainly concentrated at S4, whereas elevated TOC, TN, and NH₄^+^ contents in subsurface sediments were observed at S5, indicating localized enhancement of substrate availability and nutrient accumulation. Sediment enzyme activities showed no consistent vertical increase or decrease, but instead exhibited a distinctly patchy distribution. In surface sediments, the highest activities of all four enzymes were concentrated at S4, with S6 showing secondary high values. In subsurface sediments, elevated enzyme activities were mainly observed at S5. This pattern suggests that organic nitrogen hydrolysis, organic matter oxidation, and associated enzyme-mediated processes were governed primarily by localized substrate availability, moisture status, and microenvironmental conditions, rather than by depth-dependent variation alone (Figure 3B). GHG production rates further revealed clear gas-specific patterns (Figure 3C). CO₂ production remained consistently high across sediments, suggesting that organic carbon mineralization represents a relatively widespread biogeochemical process in the Niya River Basin. By contrast, CH₄ and N₂O exhibited stronger hotspot-like distributions. Elevated CH₄ production in surface sediments was mainly observed at S4 and S6, whereas subsurface CH₄ production increased sharply at S5. High N₂O production was primarily concentrated in surface sediments at S4, S7, and S8. Taken together, sediment biogeochemical processes in the Niya River Basin were not characterized by simple vertical stratification. Instead, under a sandy depositional setting, they showed pronounced spatial heterogeneity arising from local physicochemical variability, fine-particle-size accumulation, enhanced enzymatic activity, and discrete GHG production hotspots.

### 3.2 Influence of sediment variations on enzyme activities and GHG production rates

**Figure 4:**
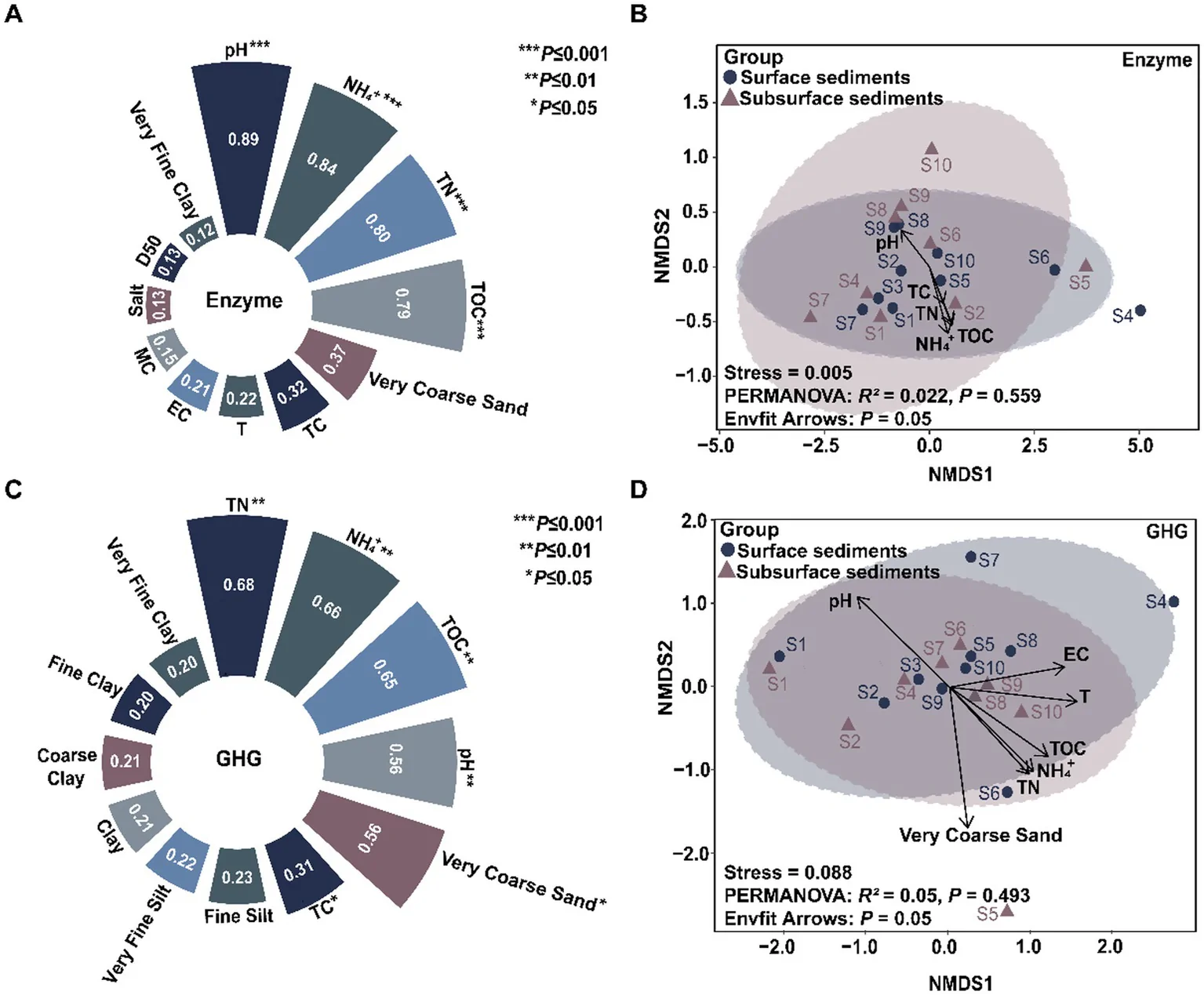
Environmental associations of sediment enzyme activity and GHG production rates. **(A,C)** Show the Mantel test results for enzyme activity and GHG production rates, respectively, with bar length representing the absolute value of Mantel’s *r* and color indicating significance levels. **(B,D)** show NMDS ordinations with fitted environmental vectors for enzyme activity and GHG production rates, respectively. Blue circles and purple triangles denote surface and subsurface sediments, and ellipses represent the distribution ranges of the two depth groups. Arrows indicate the direction and strength of environmental gradients fitted to the ordination space. The results indicate that variations in enzyme activity were strongly associated with pH, TOC, TN, and NH_4_^+^, while GHG production rates was jointly associated with carbon–nitrogen availability, pH, EC, temperature, and very coarse sand content.

Together, the Mantel test and NMDS analyses showed that spatial variation in sediment enzyme activities and GHG production rates in the Niya River Basin was not mainly driven by surface–subsurface differentiation. Rather, these patterns were jointly modulated by carbon–nitrogen nutrient availability, pH conditions, and local particle-size structure. For sediment enzyme activities, pH exhibited the strongest association, with Mantel’s |r| reaching 0.89, followed by NH₄^+^, TN, and TOC, with |r| values of 0.84, 0.80, and 0.79, respectively, and all were highly significant. However, most particle-size fractions did not display consistent significant relationships (Figure 4A). These results suggest that variations in enzyme activities were closely associated with sediment acid–base conditions and carbon–nitrogen substrate availability. Mantel analysis indicated that overall variation in GHG production rates was associated with TN, NH₄^+^, TOC, and pH. However, individual gases may exhibit different environmental associations because CO₂, CH₄, and N₂O are generated through distinct microbial pathways and respond differently to sediment redox conditions and substrate availability. Therefore, gas-specific Mantel analyses were further conducted to evaluate their individual environmental associations (Supplementary Figure S2). Gas-specific analyses further revealed divergent environmental controls among individual gases (Supplementary Figure S2). CO₂ production showed relatively broad associations with carbon–nitrogen substrates, whereas CH₄ exhibited stronger relationships with TOC, NH₄^+^, pH, and very coarse sand, suggesting enhanced sensitivity to localized substrate enrichment and sediment microhabitat conditions. N₂O showed stronger associations with nitrogen-related variables, suggesting potential links with nitrogen transformation processes. Very coarse sand was also significantly associated with CH₄ and N₂O production patterns, suggesting that coarse-grained sediment structure may potentially influence microscale habitat conditions. However, the underlying physical mechanisms require further validation through direct measurements of pore structure, oxygen availability, and redox conditions (Figure 4C). NMDS ordination further corroborated this pattern, showing a satisfactory ordination fit and supporting the robustness of the results (Stress < 0.1). Nevertheless, PERMANOVA results were not significant for either matrix, with surface–subsurface grouping explaining only 2.2 and 5.0% of the variation, respectively. These results indicate that vertical stratification exerted a weaker influence than site-scale spatial heterogeneity on sediment enzyme activities and GHG rates (Figures 4B,D). NMDS results indicated that enzyme activities were primarily separated along gradients of TOC, NH₄^+^, TN, pH, and TC, whereas GHG was jointly associated with TOC, TN, NH₄^+^, pH, EC, temperature, and very coarse sand. Enzyme activities were generally positively related to TOC, TN, and NH₄^+^, but tended to show negative associations with pH. By contrast, relationships between GHG and environmental variables varied more strongly among gas types and sediment layers (Supplementary Figure S1), highlighting the coexistence of widespread CO₂ mineralization and localized CH₄ and N₂O production hotspots. Taken together, sediment biochemical processes in the Niya River Basin were not governed by sediment depth alone. Rather, they exhibited complex spatial response patterns shaped by carbon–nitrogen substrate availability, alkaline environmental constraints, and particle-size structure differentiation.

### 3.3 Layer-specific environmental controls on enzyme activities and GHG production rates

**Figure 5:**
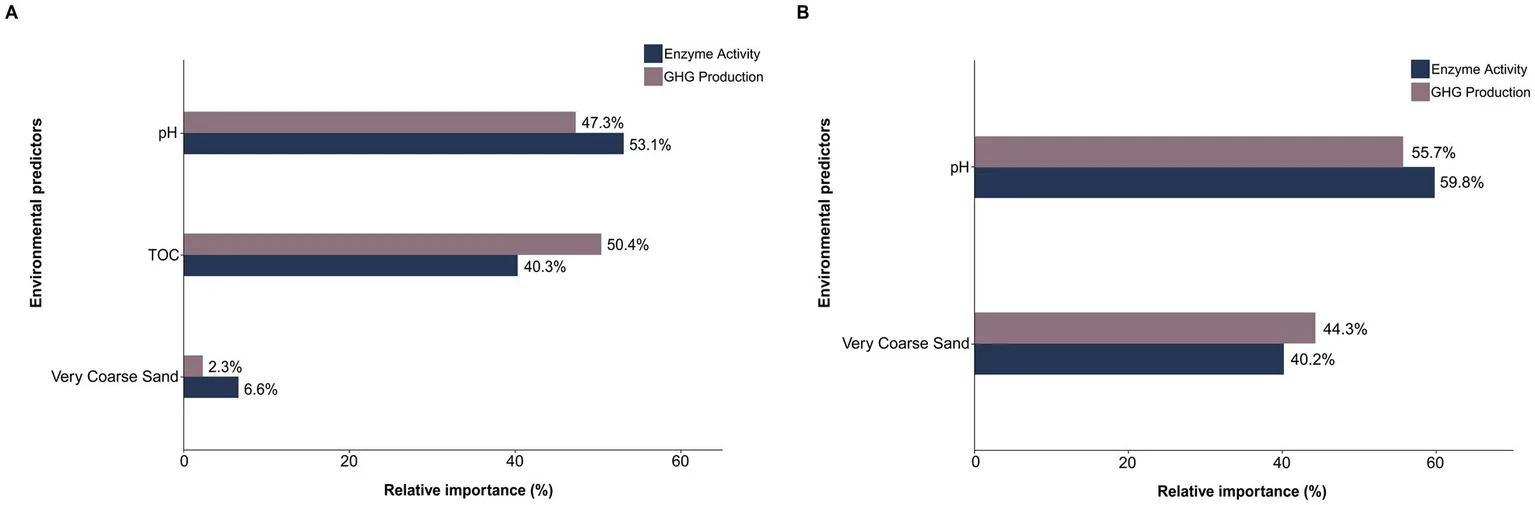
Random forest-based ranking of environmental predictors associated with enzyme activity and GHG production rates in surface and subsurface sediments. **(A)** Surface sediments. **(B)** Subsurface sediments. Predictor importance was evaluated using mean decrease in impurity, and the analysis was repeated using bootstrap resampling to assess the stability of importance estimates. The results represent relative contributions of retained predictors after collinearity screening.

Predictor selection was performed based on variance inflation factor (VIF<6) analysis to reduce multicollinearity (Supplementary Table S3), and bootstrap resampling was applied to assess the stability of variable importance estimates (Supplementary Table S4). The random forest results revealed distinct depth-dependent environmental associations on sediment biogeochemical processes (Figure 5). In surface sediments, enzyme activity was mainly associated with pH and TOC, which contributed 53.1 and 40.3% of the relative importance, respectively, whereas very coarse sand showed a limited contribution (6.6%). Similarly, GHG production rates were primarily related to TOC and pH, accounting for 50.4 and 47.3% of relative importance, respectively. These results suggest that variations in surface biogeochemical processes were closely associated with organic carbon availability and sediment physicochemical conditions. In subsurface sediments, pH showed the highest relative importance for both enzyme activity and GHG production rates, contributing 59.8 and 55.7%, respectively. Meanwhile, the contribution of very coarse sand increased markedly, accounting for 40.2% of enzyme activity and 44.3% of GHG production rates. This enhanced contribution of particle-size structure in deeper sediments suggests that sediment texture may be increasingly associated with variations in subsurface microbial processes and GHG production rates. Overall, the random forest analysis suggests that sediment biogeochemical processes exhibit depth-related environmental associations. Surface sediments were primarily characterized by stronger links with carbon availability and chemical conditions, whereas subsurface sediments showed increased associations with coarse-grained sediment structure. These findings highlight the potential importance of potential microscale sediment heterogeneity and GHG production patterns in arid sandy river sediments.

## 4 Discussion

### 4.1 Heterogeneous biogeochemical functions in the sediment-microenvironment context

The results of this study indicate that the surface and subsurface sediments in the Niya River Basin are predominantly sandy, with an average sand content of nearly 80%, confirming a typical coarse-grained depositional environment. However, under this sandy background, sediment enzyme activity and GHG production rates did not exhibit spatial homogeneity. Distinct biogeochemical hotspots occurred at a limited number of sites. These patterns indicate that sediment biogeochemical processes were not determined solely by bulk grain-size composition, but were closely associated with spatially heterogeneous carbon and nitrogen inputs linked to oasis agriculture and hydrological redistribution. Oasis agricultural zones act as key external sources of organic matter and nutrients, promoting localized enrichment and forming discrete hotspots within otherwise low-organic sandy sediments. In such environments, additional controls—including fine-particle accumulation, hydrodynamic sorting, and microenvironmental variability—further modulate substrate retention and reactivity. Owing to weak adsorption capacity, high permeability, and enhanced oxygen diffusion, sandy sediments generally exhibit limited organic matter retention. Therefore, carbon and nitrogen substrates are unlikely to form continuous, spatially uniform organic-rich sediment layers. Instead, they preferentially accumulate in localized zones where hydrodynamic energy is relatively low, fine particles are effectively retained, and moisture conditions are more favorable. Such spatially restricted substrate distribution suggests that the observed heterogeneity in enzyme activity and greenhouse gas production rates in the Niya River Basin cannot be explained solely by sediment grain-size composition. Instead, it appears to be more closely associated with localized substrate enrichment, fine-grained sediment retention, variable water availability, and microscale physicochemical heterogeneity. This pattern contrasts sharply with sediments from lakes, wetlands, and low-flow rivers in humid regions, where higher water content and stronger fine-particle accumulation are often associated with greater organic matter preservation. In humid or long-term submerged environments, sediments generally exhibit higher water content and greater fine-particle accumulation, while surface organic matter input, oxygen diffusion, and redox gradients are typically more stable (Li C. et al., 2023; Huang et al., 2023). These conditions are often associated with more pronounced vertical stratification in enzyme activity, microbial metabolism, and greenhouse-gas production. Consistent with this pattern, previous studies have shown that extracellular enzyme activity in sediments is typically higher at the surface and exhibits significant depth-dependent variation. Unlike these systems, the surface and subsurface sediments in the Niya River Basin do not show a stable separation. Both NMDS and PERMANOVA analyses indicate that sediment layer grouping explains only a limited proportion of the variability, suggesting that the vertical gradient within the 0–5 cm shallow sediment profile is weaker than horizontal heterogeneity at the site scale. This pattern may be related to the hydrological instability of arid seasonal rivers, where snowmelt runoff, intermittent floods, and dry-season sediment stabilization alternately promote sediment erosion, retransport, and redeposition. This frequent physical disturbance may reduce the likelihood of developing stable redox stratification in shallow sediments, which is more commonly observed in lakes or wetlands in humid regions. Meanwhile, surface sediments disturbance, local depositional instability, and heterogeneous sediment–water interactions may influence fine-particle redistribution, organic matter retention, and moisture preservation through changes in sediment structure, surface roughness, and transport pathways (Vahedifard et al., 2024; Firoozi and Firoozi, 2024). These sediment and microenvironmental effects may create highly patchy biogeochemical reaction zones within a predominantly sandy sediment matrix.

The Niya River Basin provides an example of how arid sandy sediments can sustain localized biogeochemical activity despite generally low organic matter retention. Rivers in arid regions are typically characterized by limited organic matter retention and relatively weak microbial activity (Pang et al., 2024; Lv et al., 2026). Despite the generally limited capacity of arid sandy sediments to retain organic matter, our results show that localized sediment environments can support enhanced enzyme activities and greenhouse gas production rates. This suggests that arid river sediments should not be viewed as homogeneous, low-reactivity media. Rather, they may form discrete “reactive patches” in areas of reduced hydrodynamic energy, localized carbon–nitrogen substrate accumulation, fine-grained deposition, or enhanced moisture retention. Consistent with this, previous studies of riverbed sediments have demonstrated that sediment type can significantly influence grain size, water storage, and CO₂ and CH₄ concentrations, underscoring the close relationship between grain structure and local biogeochemical reactions. Therefore, the defining characteristic of sediment processes in the Niya River Basin is not a traditional vertically stratified reaction model, but a patch-dominated model associated with hydrodynamic sorting, substrate accumulation, fine-particle retention, and microenvironmental heterogeneity.

### 4.2 Microbial extracellular enzyme regulation of carbon–nitrogen transformation

Sediment extracellular enzyme activity represents an important functional link between organic matter decomposition, nutrient mobilization, and microbial carbon–nitrogen transformation (Li et al., 2022). In humid-region lakes, wetlands, and river sediments, enzyme activities are commonly regulated by organic matter inputs, nutrient availability, and moisture conditions (Yang et al., 2026). However, enzyme activities in the Niya River Basin did not exhibit a simple vertical pattern between surface and subsurface sediments, but showed pronounced spatial variability associated with sediment physicochemical conditions and substrate availability. In sandy sediments, weak organic matter retention and strong hydrological disturbance limit the formation of continuous resource-rich layers, whereas localized enrichment of TOC, TN, and NH₄^+^ may create discrete hotspots with enhanced enzymatic activity (Yan et al., 2025). Consistent with this pattern, Mantel analysis identified pH as the strongest environmental association with enzyme activity, and random forest analysis further highlighted pH and TOC as the major contributors across sediment layers (Figure 5; Supplementary Table S4). These results suggest that enzyme-mediated processes in this arid sandy river system were primarily associated with local carbon availability and alkaline sediment conditions rather than sediment depth alone. The strong relationship between pH and enzyme activity likely reflects the environmental filtering effect of alkaline conditions, as pH can regulate enzyme stability, substrate accessibility, and nutrient transformation processes (Naz et al., 2022).

Carbon availability further shaped the spatial distribution of enzyme activity. Previous studies have shown that enhanced organic matter inputs stimulate microbial enzyme production by increasing substrate acquisition demand and regulating carbon transformation processes (Chen et al., 2026; Yang et al., 2023). However, unlike humid-region sediments where high moisture availability and fine-particle accumulation promote relatively continuous organic matter preservation (Li Q. et al., 2023; Ward et al., 2022), sandy sediments in the Niya River Basin have limited capacity for long-term organic matter retention. Therefore, substrate–enzyme coupling was mainly expressed through localized hotspots rather than uniform enrichment across the sediment profile. Although TN and NH₄^+^ provide essential nitrogen resources for microbial growth and enzyme synthesis (Wang et al., 2026), their lower importance compared with pH and TOC suggests that nitrogen availability alone cannot explain variations in enzyme activity. Instead, enzyme-mediated carbon–nitrogen transformation likely reflects the combined influence of carbon supply, nitrogen status, and alkaline sediment environments. Overall, the Niya River Basin exhibits a patch-based regulation pattern, where localized substrate accumulation and physicochemical heterogeneity sustain microbial enzyme activity despite the generally low reactivity of arid sandy sediments.

### 4.3 Gas-specific patterns of CO₂, CH₄, and N₂O production rates under heterogeneous sediment microenvironments

CO₂, CH₄, and N₂O production rates reflect different microbial pathways and are associated with distinct environmental conditions, including substrate availability, redox conditions, and sediment properties. CO₂ production is primarily associated with microbial respiration and organic carbon mineralization, which can occur across a relatively broad range of sediment environments (Liu et al., 2023). In contrast, CH₄ production is more closely associated with the availability of labile carbon substrates, the depletion of alternative electron acceptors, and the development of reducing microsites that support methanogenic activity. Such conditions are commonly observed in permanently saturated wetlands and lake sediments, where prolonged anaerobic environments provide suitable conditions for methane production and contribute substantially to ecosystem CH₄ emissions (Murguia-Flores et al., 2023; Scott et al., 2022). N₂O production differs from CH₄ because it is mainly associated with nitrogen transformation processes, including nitrification and denitrification, which are closely related to nitrogen availability and fluctuations in oxygen conditions (Ramond et al., 2022). Thus, differences in carbon and nitrogen substrates, together with variations in sediment redox environments, may result in contrasting spatial patterns among CO₂, CH₄, and N₂O production (Bastida et al., 2021).

In the Niya River Basin, these gas-specific characteristics were reflected in their contrasting spatial distributions. CO₂ production showed relatively widespread variation among sediment sites, suggesting that organic carbon mineralization occurred across a broad range of sandy sediment environments. This pattern indicates that CO₂ production represents a relatively general carbon-processing process, supported by the widespread availability of organic substrates and suitable physicochemical conditions rather than being restricted to localized hotspots. In contrast, CH₄ production exhibited a highly localized distribution, with elevated rates mainly observed at surface sediments S4 and S6 and subsurface sediment S5. The high CH₄ production potential at subsurface S5 coincided with elevated TOC, TN, and NH₄^+^ concentrations, suggesting that localized substrate enrichment and sediment heterogeneity may provide favorable conditions for methane production within the predominantly sandy sediment matrix. This pattern differs from humid-region aquatic systems, where persistent inundation and stable reducing conditions generally promote more continuous methane production (Zhumabieke et al., 2024). N₂O production displayed another distinct pattern, with higher rates mainly occurring in surface sediments at S4, S7, and S8, suggesting closer associations with surface nitrogen availability and dynamic sediment conditions. Consistent with these observations, gas-specific Mantel analyses revealed divergent environmental associations among individual gases (Supplementary Figure S2) CO₂ showed broader relationships with carbon-related substrates, CH₄ exhibited stronger associations with TOC, NH₄^+^, pH, and very coarse sand, whereas N₂O was more closely associated with nitrogen-related variables. These results indicate that that GHG production in arid sandy river sediments does not follow a uniform pattern but instead reflects gas-specific responses to heterogeneous substrate availability and sediment conditions, with widespread carbon mineralization coexisting with localized methane production and spatially variable nitrogen cycling.

### 4.4 Potential physical pathways linking very coarse sand to subsurface GHG production rates

Our results identified a strong statistical association between very coarse sand and subsurface GHG production rates. Although the underlying physical mechanisms remain unresolved, this relationship suggests that sediment texture may be associated with subsurface biogeochemical variability through potential links with microscale physical conditions. Random forest analysis revealed that very coarse sand had a high relative importance for subsurface GHG production rates (44.3%), followed by pH (55.7%), indicating that particle-size structure may represent an important environmental factor associated with variation in deeper sediments. In contrast to fine-grained sediments, where clay and silt fractions generally enhance organic matter retention through adsorption and promote microbial colonization, coarse sandy sediments have traditionally been considered less reactive because of their limited capacity for organic matter preservation. However, in sand-dominated arid river sediments, particle-size effects may operate through different pathways. Rather than enhancing organic matter storage, very coarse sand may be associated with differences in sediment architecture, water distribution, and potential gas transport pathways, thereby contributing to spatial heterogeneity in subsurface sediment conditions. Unlike humid-region sediments characterized by persistent saturation and fine-particle deposition, arid river sediments are frequently reshaped by episodic floods and wet–dry cycles. Under such dynamic conditions, coarse-grained structures may be related to variations in the spatial distribution of water and dissolved substrates and potentially influence microscale oxygen and redox variability. These physical differences may be associated with heterogeneous reaction zones where coarse particles, localized fine-particle patches, and organic matter-enriched microsites interact to shape the production processes of CO₂, CH₄, and N₂O (Vowinckel et al., 2023; Lin et al., 2025). This pattern differs from humid-region systems, where fine-particle accumulation commonly promotes carbon preservation and anaerobic processes through organic matter adsorption and burial (Ya et al., 2022; Meng et al., 2026).

**Figure 6:**
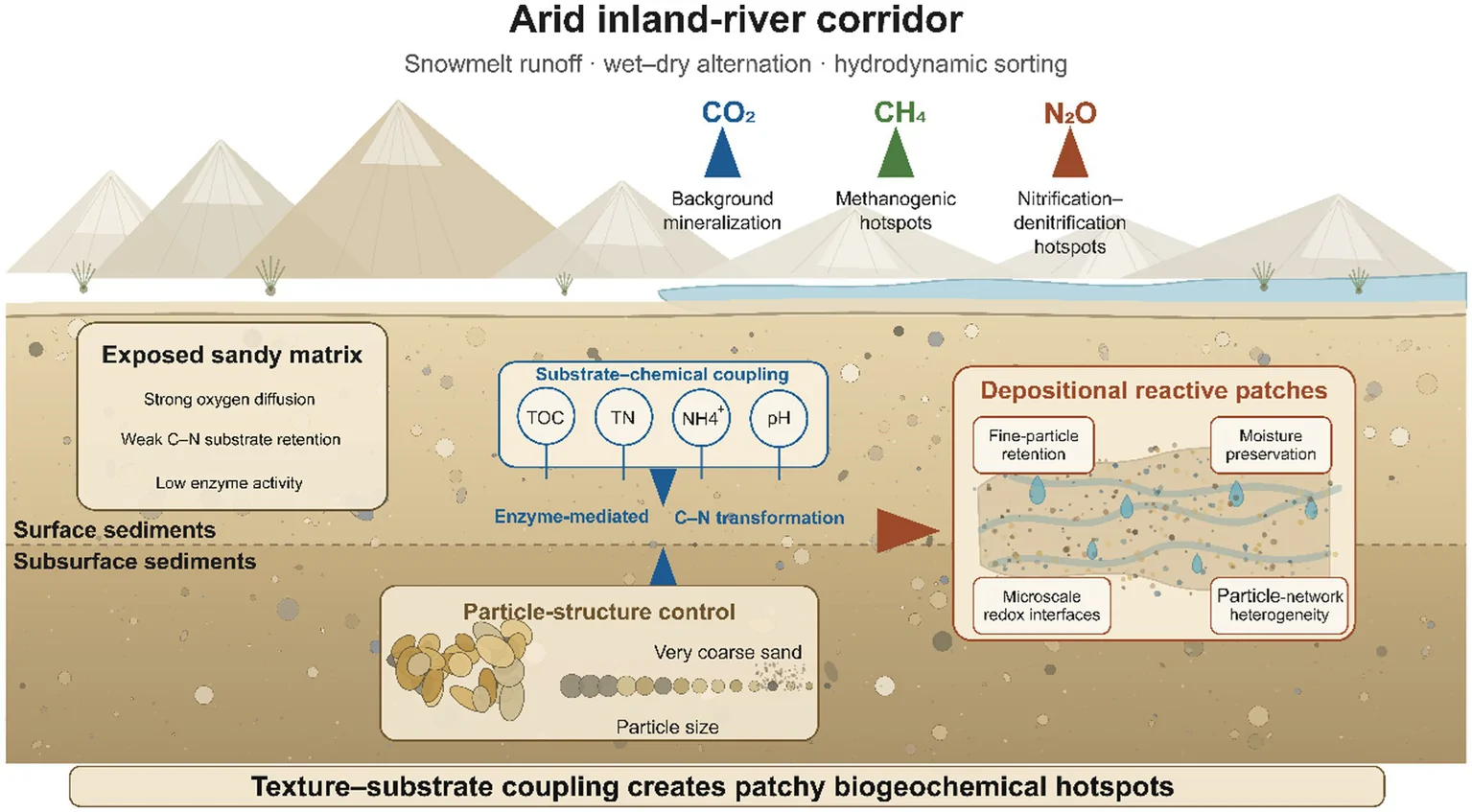
Conceptual framework of patchy biogeochemical hotspots in arid inland-river sandy sediments. Hydrological processes shape sediment texture and heterogeneity. Local substrate availability and microenvironmental conditions were associated with variations in enzyme activity and biogeochemical processes, potentially contributing to the occurrence of biogeochemical hotspots.

Based on these observations, we propose a conceptual “physical conduit effect” as a hypothesis describing how coarse-grained sediment structures may potentially be associated with subsurface GHG production by modifying particle-size connectivity, water distribution, and potential gas transport pathways. This hypothesis does not represent a confirmed mechanism, but provides a conceptual explanation for why coarse sandy sediments may contain heterogeneous biogeochemical activity despite limited organic matter retention. Future studies integrating direct measurements of permeability, particle-size network structure, moisture dynamics, oxygen availability, and redox conditions are needed to determine whether these physical pathways contribute to subsurface GHG production (Figure 6).

## 5 Conclusion

This study reveals pronounced spatial heterogeneity in sediment biogeochemical processes within the predominantly sandy Niya River Basin. Despite the generally low organic matter retention capacity of arid sandy sediments, localized enrichment of TOC, TN, and NH₄^+^ was associated with enhanced enzyme activities and GHG production rates, indicating the presence of discrete biogeochemical hotspots. Extracellular enzyme activities reflected the close linkage between substrate availability and microbial carbon–nitrogen transformation processes, with pH and TOC representing important environmental factors associated with variations in enzymatic activity. CO₂, CH₄, and N₂O production rates exhibited distinct spatial patterns, with CO₂ showing relatively widespread variation, whereas CH₄ and N₂O displayed stronger spatial localization associated with specific sediment conditions. Furthermore, the increased contribution of very coarse sand to subsurface GHG production rates suggests that sediment textural heterogeneity may be associated with variations in deeper sediment processes and potentially related to microscale environmental conditions. Overall, arid sandy river sediments should be recognized as heterogeneous biogeochemical systems, where substrate availability, enzyme-mediated microbial processes, and sediment physical structure are associated with spatial variability in carbon–nitrogen transformation and GHG production.

## Data Availability

The raw data supporting the conclusions of this article will be made available by the authors, without undue reservation.
