# Supplementary material for: Microbial enzyme-driven carbon-nitrogen cycling processes of greenhouse gases in sandy sediments of river-oasis systems in arid regions
Source: Front Microbiol. 2026 Sep 3;17:1912116. doi: 10.3389/fmicb.2026.1912116 (PMC13543408; doi:10.3389/fmicb.2026.1912116)
Supplement: Supplementary file 1 [file Data_Sheet_1.PDF]

Supporting Information

For

Microbial enzyme-driven carbon and nitrogen cycling processes of greenhouse gases in sandy sediments of river-oasis systems in arid regions

Guoqing Lv[a], Junjian Wang[b], Lijun Hou[c], Xigang Liu[a,d], Mao Ye[a,d], Xiaoshuang Zhao[a,d], Yaqi Song[a,d], Zongxiao Zhang[a,d,e*], Yonghui Wang[a,d*]

[a]College of Geography Science and Tourism, Xinjiang Normal University, Urumqi 830017, China

[b]State Key Laboratory of Soil Pollution Control and Safety, MEE Environmental Protection Key Laboratory of Integrated Surface Water-Groundwater Pollution Control, Guangdong Provincial Key Laboratory of Soil and Groundwater Pollution Control, School of Environmental Science and Engineering, Southern University of Science and Technology, Shenzhen 518055, China

[c]State Key Laboratory of Estuarine and Coastal Research, East China Normal University, Shanghai 200241, China

[d]Xinjiang Laboratory of Lake Environment and Resources in Arid Zone, Xinjiang Normal University, Urumqi 830017, China

[e]Technical Research Center for Environmental Geotechnical Engineering Restoration and Resource Utilization, Xinjiang Normal University, Urumqi 830017, China

*Corresponding author, E-mail address: 107622024010031@xjnu.edu.cn and wyhsd_3011@xjnu.edu.cn

Table S1. Average values of surface sediments physicochemical properties from the Niya River Basin

Lable	Salt	MC	TP	TK	T	DO	D50	MZ	Clay	Silt	Sand	TC	TOC	NO_2^-	NO_3^-	NH_4^+	PH	EC	TN
S1	117	60	16	39	12.2	26.1	90.34113	99.25138	4.804	27.058	68.138	23.29	1.01	0.03	0.27	0.22	9.45	68.3	0.3
S2	42	42.3	5	14	9.7	18	139.02826	185.25095	1.585	19.674	78.741	16.63	1.18	0.02	0.72	0.17	9.45	66.8	0.24
S3	137	60	19	47	23.4	22.9	306.10699	338.92526	0.487	1.561	97.952	12.02	0.82	0.02	0.21	0.63	9.56	63.2	0.18
S4	305	60	42	103	26.7	21.9	127.44306	165.51085	1.326	9.105	89.57	22.97	11.19	0.02	0.89	8.52	8.11	181.8	0.92
S5	1405	48.6	175	432	26.6	15.2	105.25181	121.6278	1.559	21.791	76.65	15.85	2.93	0.02	0.23	0.32	9.28	211.3	0.48
S6	331	27.4	44	108	26.8	15.4	93.11385	106.55509	3.65	23.544	72.806	20.48	3.8	0.02	1.51	3.57	8.42	161.7	0.45
S7	832	40.2	109	240	25.3	15.8	96.82197	97.82399	1.395	12.889	85.716	15.51	0.7	0.02	0.76	0.48	9.59	120.1	0.21
S8	538	41.4	74	183	28	24.6	129.40473	140.42948	1.555	9.255	89.19	16.53	0.81	0.02	1.76	0.33	9.65	122.1	0.2
S9	264	41.2	36	90	32.1	16.7	92.2966	90.79089	2.513	22.602	74.885	16.98	0.63	0.04	0.32	0.18	9.34	135.2	0.19
S10	709	46.6	89	220	29.7	15.8	79.06194	93.47367	4.899	34.605	60.496	20.41	1.68	0.02	0.32	0.41	9.24	138	0.28

Table S2. Average values of subsurface sediments physicochemical properties from the Niya River Basin

Lable	Salt	MC	TP	TK	T	DO	D50	MZ	Clay	Silt	Sand	TC	TOC	NO_2	NO^-	NH_4^+	PH	EC	TN
S1	117	60	16	39	12.2	26.1	124.03817	144.01423	1.323	16.268	82.409	22.64	0.91	0.02	0.21	0.41	9.58	70.6	0.23
S2	145	56	20	49	23	20.2	80.85646	90.26776	1.519	27.998	70.483	21.61	1.05	0.02	0.58	2.01	9.11	62	0.34
S4	137	60	19	47	23.4	22.9	295.40164	324.84667	0.609	1.381	98.011	9.98	0.68	0.02	0.18	0.33	9.58	69	0.17
S5	209	51.7	28	70	26.7	21.9	131.31116	189.45954	1.425	12.147	86.428	27.76	6.33	0.02	0.3	5.14	8.44	113.6	0.98
S6	1000	45.2	150	355	26.6	15.2	66.87717	76.11461	3.699	43.309	52.992	20.91	1.57	0.02	0.13	0.37	9.17	149.9	0.37
S7	2292	52.7	235	579	25.3	15.8	106.59166	109.84148	1.028	7.8	91.171	16.57	0.58	0.21	0.81	0.27	9.54	231	0.18
S8	442	46.8	64	155	28	24.6	78.29966	81.70462	2.063	29.556	68.381	16.51	0.75	0.22	0.92	0.33	9.56	97.3	0.17
S9	330	53.1	46	114	32.1	16.7	77.77609	83.74697	2.442	32.444	65.114	16.78	0.71	0.02	0.15	0.31	9.73	68.7	0.23
S10	572	45.2	78	194	29.7	15.8	133.2441	144.65699	1.919	9.1	88.981	15.57	0.79	0.08	0.5	0.25	9.17	154.2	0.23

Table S3. Variance inflation factor (VIF) values of predictors retained in random forest models

Depth	Predictor	VIF	Tolerance	Collinearity assessment
**Surface sediments**	TOC	4.92	0.2	Acceptable
	pH	5.1	0.2	Acceptable
	Very Coarse Sand	1.09	0.92	Low collinearity
**Subsurface sediments**	pH	3.1	0.32	Acceptable
	Very Coarse Sand	3.1	0.32	Acceptable

Table S4. Relative importance of environmental predictors based on random forest analysis and bootstrap resampling

Depth	Response	Predictor	Mean decrease in impurity	Bootstrap SD	Relative importance (%)
**Surface sediments**	Enzyme activity	pH	7.557	0.266	53.1
		TOC	5.736	0.270	40.3
		Very Coarse Sand	0.946	0.084	6.6
	GHG production	TOC	15.338	0.695	50.4
		pH	14.394	0.668	47.3
		Very Coarse Sand	0.691	0.193	2.3
**Subsurface sediments**	Enzyme activity	pH	6.257	0.210	59.8
		Very Coarse Sand	4.203	0.212	40.2
	GHG production	pH	14.843	0.646	55.7
		Very Coarse Sand	11.798	0.604	44.3

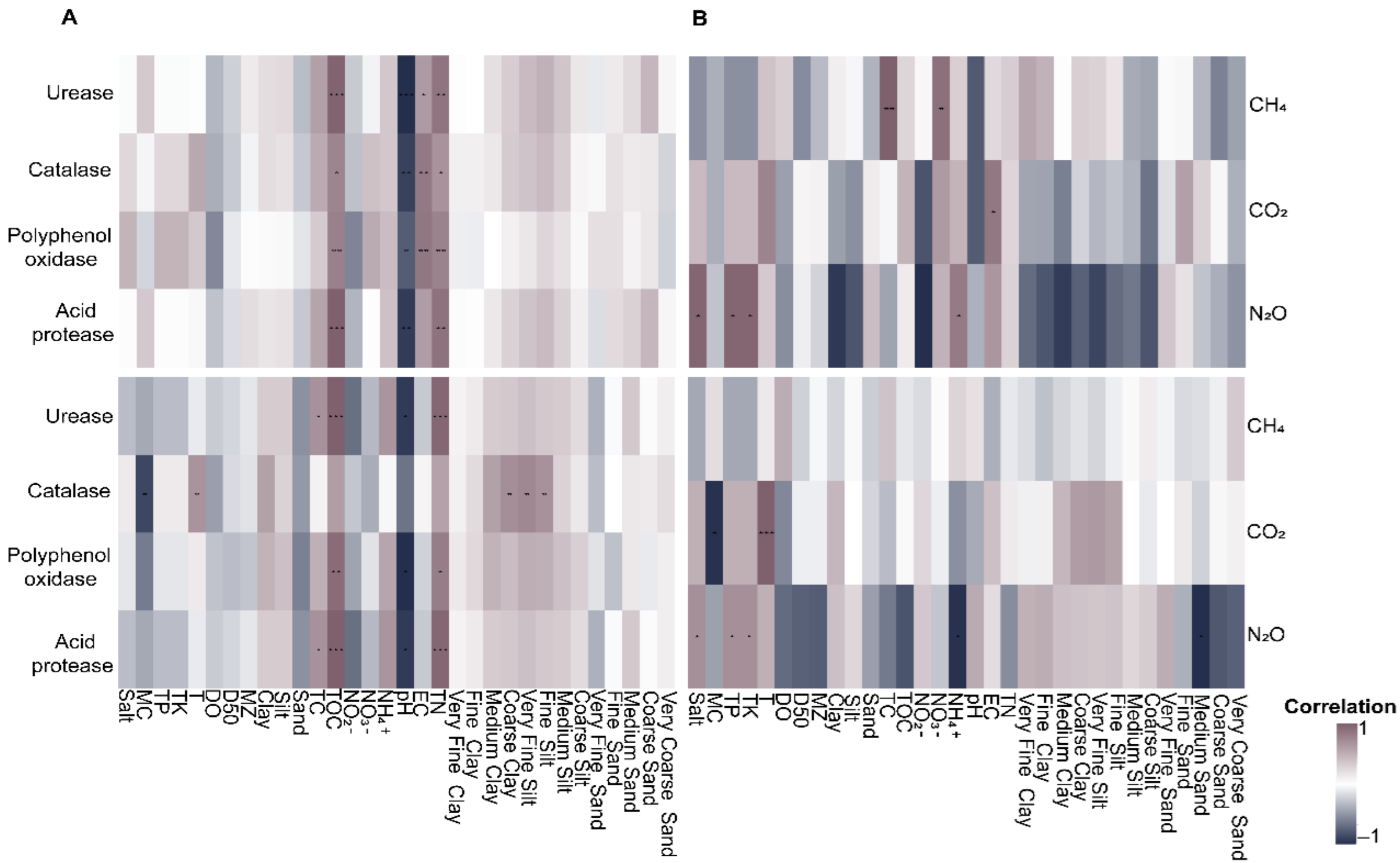

Figure S1. Spearman correlation heatmap between sediment biochemical variables and environmental factors at different depths. A, correlations between sediment enzyme activities and physicochemical properties as well as particle-size fractions; **B**, correlations between GHG production rates and

physicochemical properties as well as particle-size fractions. The upper and lower panels represent the 0–5 cm surface sediments and 5–10 cm subsurface sediments, respectively. The color gradient indicates the Spearman correlation coefficient, with warm and cool colors representing positive and negative correlations, respectively. Asterisks denote significance levels: $P < 0.05$, $P < 0.01$, and $P < 0.001$.

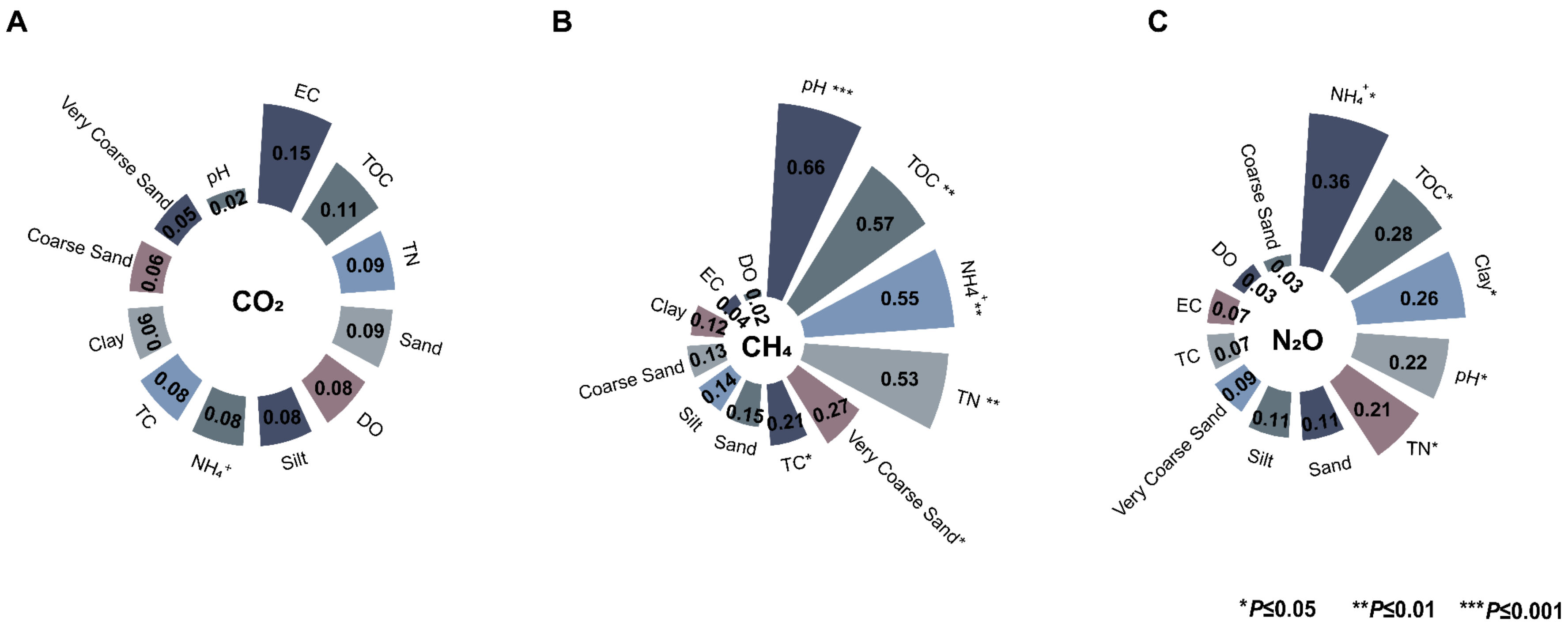

Figure S2. Gas-specific Mantel test analyses of relationships between sediment properties and CO_2, CH_4, and N_2O production rates. Mantel's r values indicate correlations between individual gas production rates and environmental variables for CO_2 (A), CH_4 (B), and N_2O (C). Asterisks denote significance levels (*P ≤ 0.05, **P ≤ 0.01, ***P ≤ 0.001). Environmental variables include sediment physicochemical properties, carbon–nitrogen substrates, and particle-size fractions. The results demonstrate distinct environmental controls on CO_2, CH_4, and N_2O production in arid sandy river sediments.
